# Towards an AI-driven soft toy for automatically detecting and classifying infant-toy interactions using optical force sensors

**DOI:** 10.3389/frobt.2024.1325296

**Published:** 2024-03-12

**Authors:** Rithwik Udayagiri, Jessica Yin, Xinyao Cai, William Townsend, Varun Trivedi, Rohan Shende, O. Francis Sowande, Laura A. Prosser, James H. Pikul, Michelle J. Johnson

**Affiliations:** ^1^ Rehabilitation Robotics Lab (A GRASP Lab), University of Pennsylvania, Philadelphia, PA, United States; ^2^ Pikul Research Group (A GRASP Lab), University of Pennsylvania, Philadelphia, PA, United States; ^3^ Department of Mechanical Engineering and Applied Mechanics, University of Pennsylvania, Philadelphia, PA, United States; ^4^ Department of Bioengineering, University of Pennsylvania, Philadelphia, PA, United States; ^5^ Department of Pediatrics, University of Pennsylvania, Philadelphia, PA, United States; ^6^ Department of Mechanical Engineering, University of Wisconsin-Madison, Madison, WI, United States; ^7^ Department of Physical Medicine and Rehabilitation, University of Pennsylvania, Philadelphia, PA, United States

**Keywords:** pediatric, rehabilitation, smart toy, robotics, infant-toy interactions, machine learning, assessment, soft force sensor

## Abstract

**Introduction:** It is crucial to identify neurodevelopmental disorders in infants early on for timely intervention to improve their long-term outcomes. Combining natural play with quantitative measurements of developmental milestones can be an effective way to swiftly and efficiently detect infants who are at risk of neurodevelopmental delays. Clinical studies have established differences in toy interaction behaviors between full-term infants and pre-term infants who are at risk for cerebral palsy and other developmental disorders.

**Methods:** The proposed toy aims to improve the quantitative assessment of infant-toy interactions and fully automate the process of detecting those infants at risk of developing motor delays. This paper describes the design and development of a toy that uniquely utilizes a collection of soft lossy force sensors which are developed using optical fibers to gather play interaction data from infants laying supine in a gym. An example interaction database was created by having 15 adults complete a total of 2480 interactions with the toy consisting of 620 touches, 620 punches—“kick substitute,” 620 weak grasps and 620 strong grasps.

**Results:** The data is analyzed for patterns of interaction with the toy face using a machine learning model developed to classify the four interactions present in the database. Results indicate that the configuration of 6 soft force sensors on the face created unique activation patterns.

**Discussion:** The machine learning algorithm was able to identify the distinct action types from the data, suggesting the potential usability of the toy. Next steps involve sensorizing the entire toy and testing with infants.

## 1 Introduction

About 5%–10% of infants are born with neurodevelopmental impairments resulting from disorders such as Cerebral Palsy (CP) (Rydz et al., 2005). These impairments often affect cognition and motor function (Shivakumar et al., 2017). Seventy to eighty percent of CP cases develop birth complications including asphyxia, and preterm birth (Vitrikas et al., 2020). While CP is a lifelong progressive disorder with no cure, effective rehabilitation can increase quality of life. Rehabilitation has been shown to be especially effective before 2 years of age, due to the high neuroplasticity seen in the infant brain (Morgan et al., 2016). In order for this early intervention to occur, it is imperative that impairments manifesting as neurodevelopmental delays are detected early in this critical period of brain development. Detecting impairments in infants is a difficult task, however. There exist clinical assessments such as the Test of Infant Motor Performance (TIMP), and General Movements Assessment (GMA) that can predict whether an infant is at a high risk of having a diagnosis of CP at 18 months (Kolobe et al., 2004). However, clinical tests tend to be less accessible in low resource settings in that they require qualitative classifications which need clinicians to undergo time consuming and expensive clinical training in order to learn how to administer correctly (Kolobe et al., 2004; Noble and Boyd, 2012; Bosanquet et al., 2013). These issues have resulted in low implementation in low and middle-income countries where children with CP have a significantly poorer health-related quality of life and increased mortality rate due to lack of accessible early detection and rehabilitation (Oguntade et al., 2022).

A potential remedy to this general lack of early and attainable testing could be the use of accessible technologies including AI and low-cost sensors to help automate processes, leading to more quantitative assessments that require less time to master. Evidence shows that AI and robotic technology systems have the potential to allow for easier, and more objective testing. For example, the Play and Neurodevelopment Asessment Gym (Ho et al., 2019; Chambers et al., 2020; Prosser et al., 2021; Kather et al., 2023) and CareToy (Serio et al., 2013; Cecchi et al., 2016; Rihar et al., 2016) are two existing technology efforts to improve early assessment of infants with neuromotor delays. These systems use pressure sensors, robotic and mechatronic toys, and machine learning to characterize infants at play with and without one or more toys. The sensor infused toys are a critical part of these early detection efforts. The CareToy (Rihar et al., 2016) and the SmarToy Gym (Goyal et al., 2017)—an early version of the PANDA Gym—are gym systems that both use a sensorized environment plus multiple smart toys to collect a variety of kinematic and kinetic data that are then used to identify predictors of neuromotor delay.

To best serve the purpose of collecting insightful and robust data on infant interaction, a smart toy should be able to perform relevant interactions with infants at different stages. When trying to understand infant development, it is common to analyze their upper and lower body movements. In their first 2–3 months, infants are known to be capable of batting toys and following certain colors (Gerber et al., 2010). At 3–4 months, they are able to kick and reach for objects (Gerber et al., 2010). Then at 4–5 months, they can reach for and grasp objects (Gerber et al., 2010). After which at 6 months they are capable of banging and shaking toys (Gerber et al., 2010). It has been shown that in comparison to their typically developing counterparts, infants born at risk exhibit less refined control of their upper and lower body functions when it comes to actions such as kicking (Heathcock et al., 2005; Deng et al., 2018), reaching (van der Heide et al., 2005; Guimarães et al., 2013), and grasping (de Almeida Soares et al., 2014). With an understanding of infant milestones and how their performance differs between typically developing and at-risk infants, it can be concluded that a smart toy that can distinguish these groups should be capable of identifying the various interactions seen at these milestones. This includes being able to quantify grasp, contact for when the infant reaches for the toy, and impact from kicking and batting.

The smart toys used in the CareToy, SmarToy Gym and PANDA Gym are designed to elicit infant actions such as gazing, reaching, grasping and kicking. The toys use sound and/or flashing lights to encourage infants to engage. Accelerometers, gyroscopes, and pressure sensors embedded in the toys then capture the interactive data. A limitation of these toys is their inability to automatically differentiate between the types of physical interactions the infant imparts to it. A corresponding video capture and analysis is often required to confirm the type of infant interaction (Kather et al., 2023).

In a data-driven world, collecting rich data to analyze risk patterns is critical. Since risk patterns can give insight into an infant’s neurodevelopment, if these patterns are identified and treated early, then infant functional outcomes improve. This paper describes the design and development of a smart toy that uniquely utilizes a collection of soft lossy force sensors which are developed using optical fibers to gather and classify physical interaction data such as touches, punches, and grasps. We describe the creation of a database of interactions with the toy and a machine learning model developed to classify the interactions present in the database. The machine learning algorithm was able to identify the distinct action types from the data, suggesting the potential usability of the toy.

### 1.1 Previous work

**FIGURE 1:**
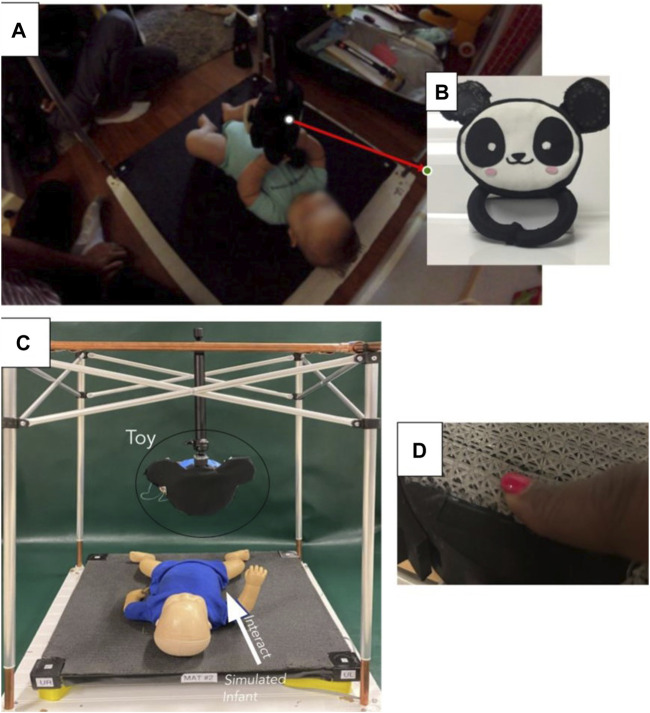
**(A)** Infant playing with the Ailu toy in the PANDA gym **(B)** The original Ailu toy (Ho et al., 2019; Chambers et al., 2020; Prosser et al., 2021), **(C)** Infant playing with the revised Ailu toy in a revised version of the PANDA gym **(D)** Human touch showing compliance of lattice structure.

The Play and Neuro Development Assessment (PANDA) gym is an infant play gym created as a tool for collecting quantitative metrics for detecting impairment in infants as young as 1–6 months (Goyal et al., 2017). Figure 1A shows the current PANDA GYM with an infant. The PANDA Gym system collects these metrics through the use of multiple cameras, a pressure mat, and the sensorized Ailu toy (Ho et al., 2019; Chambers et al., 2020; Prosser et al., 2021). The Ailu toy (Ho et al., 2019) is a critical part of the PANDA Gym and the first version shown in Figure 1B demonstrates proof of concept to collect relevant data reliably. Repeated use of the toy revealed shortcomings and inconsistent data capture.

The original Ailu toy collected data primarily through specially developed force-sensing resistors (FSRs) to detect contact at its ears and face, and pressure sensors to detect squeezing at its arms. Although the FSRs collected analog data, due to their poor sensitivity, the data could only be processed as a binary interaction, simply indicating whether the ears or face had been touched. Due to this shortcoming of the FSRs, insight into how hard the infants touched or hit the face and ears was lost.

The FSRs of the Ailu toy led to another shortcoming, as they were inconsistent in detecting contact with the infant. In order to have FSRs large enough to encompass both the face and ears of the toy, they had to be custom-developed. They consisted of two layers of conductive fabric with a layer of pressure-sensitive velostat sheet in between them. With this configuration, whenever the sensor was pressed, all three sheets came in closer contact with each other, allowing for a change in resistance. To measure this change in resistance, the two layers of the conductive fabric were each connected to a wire via conductive thread. Although this configuration was verified to be capable of collecting contact data, it was not robust to repeated use. The pressure-sensitive velostat sheet could not withstand the sometimes rough interactions with infants and would tear and the conductive thread that allowed for interface with the two layers of conductive sheet would come loose. As a result, the Ailu toy inconsistently recorded data from its face or ears, which served as major areas of interaction, and data often had to be manually verified through visual analysis of camera recordings of the data collection sessions. In response, the proposed smart toy, Ailu 2, upgrades the existing solution by leveraging the latest trends in Soft Robotics, Soft Sensing, Machine Learning and Medical Device design. The primary physical change planned is to incorporate Soft Force Sensors, fabricated using low-cost optical fibers and waveguides, to make a compliant toy that is easy for infants to interact with as compared to the existing rigid FSR sensors.

The use of optical fibers and waveguides to detect touch, interactions, strain, Proprioception, Exteroception, and force-sensing has been explored (Xu et al. (2019); Zhao et al. (2016). Both Xu and others and Zhao and others demonstrate the usefulness of the optical fibers and custom waveguides for the detection of touch, interactions, pressure, strain, proprioception, exteroception and force sensing. Zhao and others showed its utility within a soft prosthetic hand. The key principle for accurate sensing is the ability to detect and quantify the amount of light loss through the optical fibers due to absorption based on Beer-Lambert’s law,

A=eLc (1)

where *A* is absorbance, *L* is the path length, *e* is the material’s absorptivity, and *c* is the concentration of chemical species in the medium that attenuates light. This work showed the relation between elongation and power loss in waveguides and their repeatability. Proprioception and exteroception are observed by waveguides interacting with each other and causing light to escape from one and be transferred to the other and detecting the captured light at the receiver end. To and others (To et al., 2015) showed a use case with highly stretchable fibers. They characterized pressure and strain to light loss due to cracks in the protective coating that occur due to elongation or bending of the fibers. They demonstrated that a gold reflective layer could decrease light loss during transmission and form micro-cracks on bending, strain, and elongation. Instron testing and characterization showed promising results for this soft optical strain sensor. Although the results are promising, the fabrication of a waveguide coated with a gold layer is expensive and time-consuming. Several articles (Missinne et al., 2014; Harnett et al., 2017; Leber et al., 2019) illustrate the fabrication of cost-effective stretchable optical fibers that can undergo multiple strain cycles with repeatable transmission and attenuation behavior. The experimentation included stretching, bending, and indenting the optical fiber and observing the change in transmission for multiple wavelengths. The trend observed with this experimentation follows the Beer-Lambert Law, Equation 1 that strengthens our hypothesis of an optical force sensor based on the property of light lost due to bending. There has been a lot of work in medical devices for rehabilitation that uses force sensors to measure and observe pressure patterns. Bobin et al. (2018) demonstrates a smart cup for upper limb rehabilitation, while Mennella et al. (2021) is a review of technology aided hand assessment in which FSRs show maximal use. Our proposal of a lossy sensor that works similar to a force sensor but is capable of collecting richer data can have a wider use case in applications mentioned in these papers (Bobin et al., 2018; Mennella et al., 2021). Barlow et al. (2020) uses strain gauges to collect force data and discusses in depth data analysis of these collected force signatures. The paper talks about different statistical values like SD, peak force, dF/dtmax, etc. which conveys significant information. We draw motivation from this and plan to use deep learning to learn these statistical values and understand underlying patterns. In the following section, we describe our development of a cost-effective force sensor using commercially available products and 3D printed parts.

## 2 Methods

### 2.1 Lossy sensor toy development

Ailu2 Toy is being developed with the intention to replace all force and pressure sensors in Ailu Toy with the novel lossy force sensors developed using optical fibers. Figure 1 summarizes the current stage of development of the Ailu2 toy and how it is intended to be used with the PANDA Gym as a drop in for the original smart toy (Figure 1B). Figure 1C shows a simulated infant in the gym structure on the pressure mat and the arrow represents the interaction between the infant and the toy—only the face and support structure has been sensorized. Figure 1D shows the compliance of the lattice structure housing the optic fiber and emphasizing the “soft” aspect of the proposed toy. The development of the single sensor and driving electronics is discussed in Section 2.1.1.

#### 2.1.1 Lossy sensor

**FIGURE 2:**
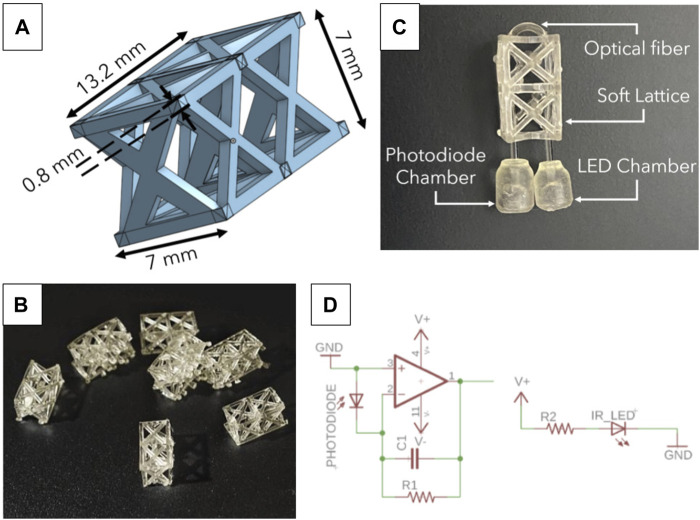
**(A)** CAD of lattice structure with dimensions **(B)** Bunch of freshly cured 3D printed lattices. The lattices are printed on a FormLabs 3 printer using a soft resin material (Flexible 80A) and cured for 10 min under UV light exposure. **(C)** Zoomed in image of the sensor with individual components labelled; this image also shows the optical fiber weave through the lattice. **(D)** Electronic circuit for single lossy sensor with components: R1 = 1M Ω, R2 = 220 Ω, C1 = 4,700 pF, V = 5 V.

The electronics and lattice structure make the core components and lay the foundations of the sensor design process. Figure 2 shows the lattice and the circuit for a single lossy force sensor. The lattice is a custom-designed structure to house an optical fiber. The lattice structure was carefully chosen to stabilize the optical fiber, inspired by the design used for the Optical Lace paper, Xu et al. (2019). The optical fiber needs to be enclosed in a structure that allows for fairly equal force distribution when compressed to ensure uniform bending. Since the diameter of the fiber is very small, the lattice must prevent the fiber from translating laterally within its structure and cause it to bend when the lattice structure is compressed. In addition, the lattice structure must ensure that the fiber bending direction is consistent and repeatable. To get accurate and reliable results the optical fiber was constrained within a lattice that uses a truss structure. The truss ensures that the force is spread evenly, that the optical fiber is restricted to bend in a particular direction, and that uniform bending is observed. Notches within the structure allow the fiber to be held in place over multiple compression cycles without the fiber moving. Figure 2A shows the computer-aided model of the lattice structure with dimensions to depict the scale and intricacy of the design. Figure 2B shows the soft resin-based 3D printed parts that were printed and cured for 10 min under UV light. Flexible 80A was the soft resin chosen to fabricate the lattice structures. With an ultimate tensile strength of 8.9 MPa (Datasheet), this material exhibits a characteristic ability to withstand moderate tensile stresses before experiencing failure. The relatively low value of 8.9 MPa signifies that Flexible 80A can deform and stretch under moderate tensile forces, ensuring that it can securely house the optical fiber while still permitting controlled compression. This characteristic makes it an appropriate choice to facilitate uniform bending of the fiber without risking structural damage. This feature is expected to enhance the reliability of the toy in assessing infant interactions and potential motor delays as we expect that its elastic property would ensure an extended lifetime with repeatable results.

Figure 2C shows the lattice and optical fiber combination. The combination of optical fiber and lattice waveguide is the **Lossy Force Sensor**, which exploits the property of light loss and correlates it to the force applied. Multiple iterations of the lossy sensor followed the initial prototype; these iterations tested varying supports, sizes, dimensions, and uses. The “Lossy” principle was still applicable irrespective of the changes made to the initial prototype.

The electronic circuit (Figure 2D) for each lossy sensor consists of an amplifier for the photodiode signal and an IR LED emitter circuit. The photodiode signal is amplified to make this signal readable by a microcontroller (Teensy 4.1). The values of the components were chosen after experimental trade-offs in sensitivity to light loss and the magnitude of voltage change. These were R1 = 1M Ω, R2 = 220 Ω, C1 = 4,700 pF, V = 5 V.

To make testing robust, and repeatable, a printed circuit board (PCB), shown in Figure 5B, was designed to contain 12 sensing circuits, which accommodates 12 lossy force sensor circuits. The Ailu2 Toy design will ultimately consists of 6 lossy force sensors for the face (shown in Figure 5C), 2 lossy force sensors for right ear, 2 lossy force sensors for left ear and 2 arm sensors.

### 2.2 Lossy sensor testing

**FIGURE 3:**
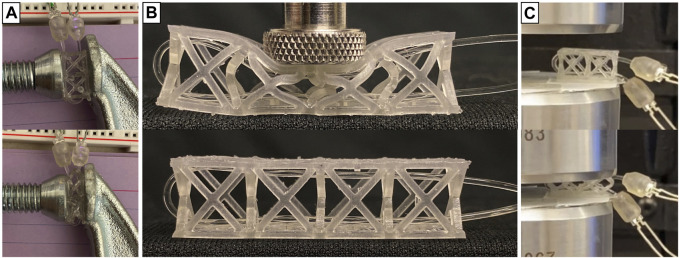
**(A)** Single sensor test bench using a C clamp with and without Clamp compressing the lattice with a fixed number of screw revolutions for repeatability **(B)** Single sensor applied force characterization using Force transducer. **(C)** Single sensor applied force characterization using Instron.

The lossy sensor was developed individually as a stand-alone force sensor that can be used in a variety of applications. Once manufactured and assembled, the lossy sensors were tested manually for different human forces and used similar to a Force Sensitive Resistor (FSR). Figure 3A shows a single lossy sensor under compression using a c-clamp. Figure 3B shows the lattice in its neutral position and when being pressed down with a fixed force. The truss structure distributes the concentrated force and causes the optical fiber inside it to bend uniformly in the direction of the force being applied. The sensor characterization process for the lossy force sensors involved subjecting single sensors alone and multiple sensors, configured as in the toy face (Figure 1E), to repeated normal loads (upto 50 N) in order to identify the limitations of this sensor.

Test bench setups with varying force ranges and data collection methods were experimented with, as shown in Figure 3. Testing of a single lossy force sensor used two procedures: 1) manual testing with a force transducer (Figure 3B) and 2) automated testing with an Instron device attached with a load cell (Figure 3C). These experiments led to the understanding of how the sensor functions under various circumstances and how the parameters-amplification gain, lattice beam thickness, color of LED, etc. can be tuned to get the desired outcomes. The signals generated from one sensor were consistent over multiple trials and different interactions.

**FIGURE 4:**
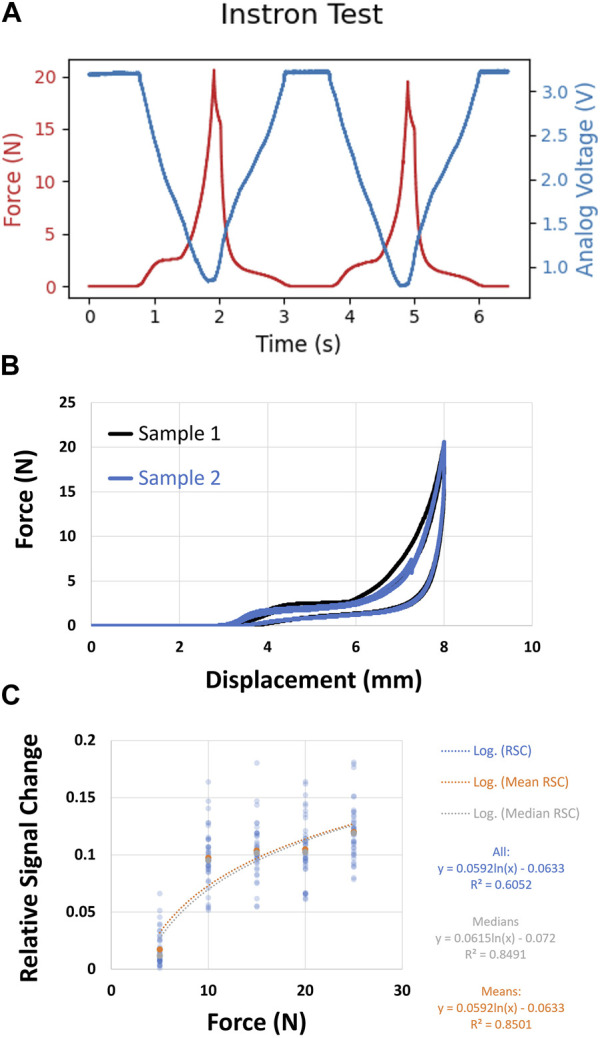
**(A)** Single sensor characterization using Instron. Red is Force (N) and Blue is Analog Voltage (V). **(B)** Hysteresis seen in the force-displacement curve as generated from Instron testing **(C)** Single sensor applied force characterization using Force transducer showing the correspondence between Analog Values and Force.

Figure 4A shows how the analog value changes when the force is applied and its corresponding force values from the Instron. Figure 4B clearly shows a hysteresis in the force-displacement graph that primarily arises due to the plasticity of the material being used. This is one of the reasons why a regression between the force applied and the analog value is difficult to determine. However, repeated testing with a handheld force transducer gave us consistent results. As seen from Figure 4C this sensor has a correspondence between the force applied and analog voltage that implies light loss. The relationship was essentially logarithmic, modeled by the following equation:

$$y=0.0592*ln\left(x\right)-0.0633$$

where *y* is the relative analog voltage change and *x* is the force applied in Newtons.

### 2.3 Lossy force sensor integration into toy

**FIGURE 5:**
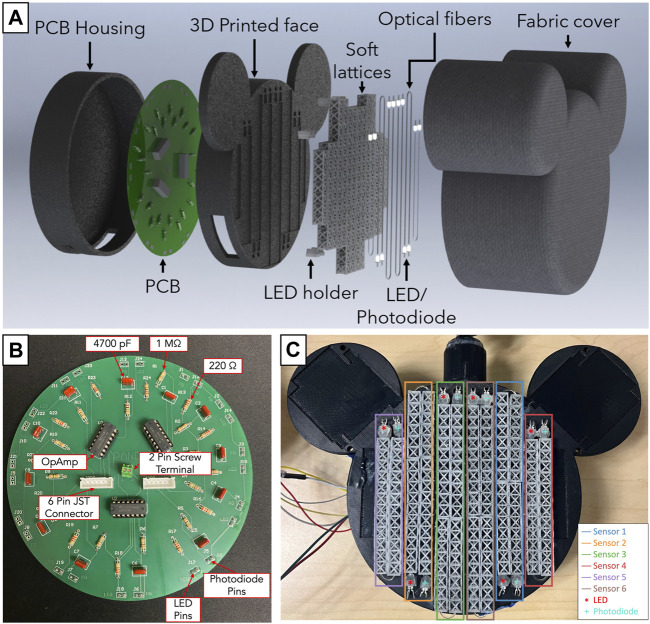
**(A)** Exploded CAD view of the toy showing each individual component that makes up the toy **(B)** Labelled PCB showing the circular arrangement for 12 sensor circuits around the circumference for ease of lattice placement across the toy **(C)** Internal arrangement of the lattices on the face of the toy. This figure shows the pair of photodiode and LED housing for each sensor.

Previous work has shown us how sensors can be integrated into a toy that is meant for infant handling. Serio and others successfully developed novel wireless toys to measure infants’ uni-manual and bi-manual actions (Serio et al., 2012; Serio et al., 2013). The previous Ailu Toy for the PANDA Gym was embedded with an inertial measuring unit (IMU), pressure sensing soft hands, and force sensing resistors for the face and ears. In this iteration of Ailu2, the face is the first to be sensorized with our custom lossy force sensors. Figure 5A shows the individual components that make up the toy: the 3D printed face, PCB and PCB housing, lossy force sensors and a infant friendly fabric cover made from poly.

The face of the toy was designed to accommodate 6 lossy sensors securely. Specifically, slots were strategically incorporated into the face to house the sensors, which were instrumental in capturing data and facilitating interaction. Slots were also made to hold the LED and Photodiode holders in place, with holes to pass wires running from the PCB to the LED and photodiodes. The face was designed using SolidWorks, and 3D printed in ABS material using Stratasys F120 FDM printer. New and longer lattices were designed such that fiber optic cables could pass exactly through their center close to the top face of the lattice, to get high sensitivity to compression. The longer lattices were designed on Onshape, and 3D printed in Flexible 80A resin using Formlabs Form 3B + Resin printer. 3D printed resin holders, placed in their respective slots, were designed to secure the LED and photodiode pairs in place on the printed face and to ensure accurate positioning and alignment of the LED and photodiode with the optical fibers.

The lossy sensors, consisting of LED and photodiode pairs, were carefully assembled. Optical fibers were woven through lattices super-glued onto the toy’s face and each end was squeezed into the holder for the LED and, then the Photodiode. The holders were hot-glued to the toy’s face to align the holes with the fibers exiting the lattices. Each photodiode and LED pair was connected to the PCB. The new lattice arrangement along with the photodiodes and LEDs are shown in Figure 5C. Figure 5B shows the PCB board housing 12 sensor components arranged symmetrically along the circumference. This arrangement allows the board to be easily populated and simplifies the routing of LED and photodiode cables to the face surface for varying lattice patterns. A separate housing, 3D printed in ABS material using Stratasys F120 FDM printer, was designed to be attached beneath the face to accommodate the Printed Circuit Board (PCB) assembly and provide structural stability to the toy. In the future, the head will accommodate the hands of the toy (Figure 5). To enhance the toy’s aesthetics and minimize the impact of ambient light on the sensors, an infant friendly fabric covering was used.

The toy force range was measured across the face using an Analog Force Gauge with a least count of 2.5*N*. The minimum and maximum force measured for each sensor shows a neat correlation with its length as shown in Table 1. The longer sensors have a larger range and this trend is continued with medium and shorter length sensors also.

**TABLE 1:** Minimum/Maximum force range for each sensor showing correlation with length of lattice.

Sensor number	Lattice length (mm)	Minimum force(N)	Maximum force(N)
1	98	2.5	40.0
2	98	5.0	45.0
3	112	5.0	52.5
4	70	2.5	30.0
5	70	5.0	25.0
6	112	5.0	47.5

### 2.4 Human interaction data collection

**FIGURE 6:**
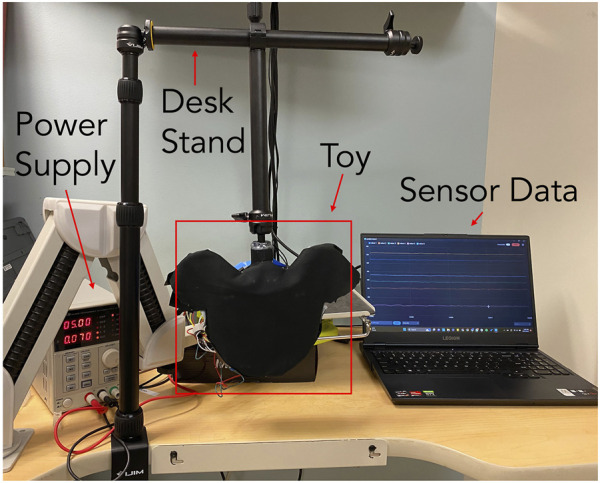
Experimental setup used with desk stand to suspend toy and collect data.

To test the viability of the lossy sensors as a tool for classifying infant-toy interactions in the PANDA gym, the toy was mounted to a VIJIM LS11 desk stand, which was, in turn, clamped onto a desk (Figure 6). The desk stand suspended the toy in an almost fixed position in space, with a little rotation at the connecting joint permitted to simulate the use-case conditions. In the PANDA gym environment, the toy would be suspended above the baby by being attached to the upper frame, with a mild degree of rotation permitted as shown in Figure 1C. Thus, this setup enabled the emulation of the PANDA gym set-up while maintaining ease of access for subject trials. Additionally, a power supply set to 5 V was connected to the toy.

Fifteen adult subjects helped us build a database of interactions. Adults were seated in front of the hanging toy, and then, instructed to perform the four relevant infant-toy interactions—touch, kick (replaced by a punch), weak grasp, and strong grasp. The four interactions were selected based on prior work conducted (Kather et al., 2023). In place of kicking the face, however, subjects were instructed to punch the toy to emulate a baby’s kick. No detailed instructions were given on performing the interaction—each interaction depended on the subjects’ interpretations. Subjects performed each interaction on the toy’s face 50 times each, for a total of 200 interactions per subject or 750 trials per interaction. A Python program guided the subject through the interactions while the toy collected data from the six lossy sensors. Only data on force interaction was collected and used in the database. No identifiable data was used.

### 2.5 Classification/learning algorithms

**FIGURE 7:**
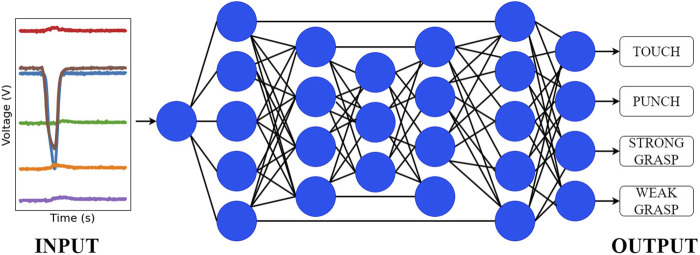
Representation of the neural network architecture derived from ResNet-50 used for classification.

Since rehabilitation of developmental disorders is especially effective before age 2, detecting impairments at an early age is important. Machine learning methods provide an efficient and accurate way to classify toy-subject interactions. We hypothesize that interactions with the toy can be classified using neural networks. Because the start and end times of each interaction were easily identified when pre-processing the data and we assume that the probability of an interaction occurring is independent of the previous one, we believe that a CNN architecture is suitable for our classification task. Additionally, compared to other network architectures like transformers, CNNs require less data to achieve accurate classification. In this study, the ResNet50 architecture (Figure 7) was used because it is a high-performing pre-trained CNN, well-suited for the relatively small PANDA data set. The dataset was pre-processed prior to training. The start time of each trial was found using Z-score thresholding and padded at the beginning of each trial with 10 values. Each trial was then clipped to 4 s to produce uniformly sized data samples for training. After this, data was normalized so that values were between 0 and 1.

To train the model on the PANDA data set, input and output dimensions in the ResNet50 model had to be modified. The input layer dimension was changed to fit 1 interaction (trial) at a time. The ‘channels’ dimension was increased to 6 for the 6 sensors contained in the toy configuration. The output layer was altered to classify four classes of data, corresponding to each type of toy-subject interaction. Before beginning the training of the model, data was split into a training set and a validation set with a ratio of 75:25. Training data (75 percent) was shuffled randomly before being fed into the model so that the trained model would not base its predictions on the order of the trials. The validation data (25 percent) was used after training the model to evaluate its performance and assess model accuracy.

## 3 Results and discussion

**FIGURE 8:**
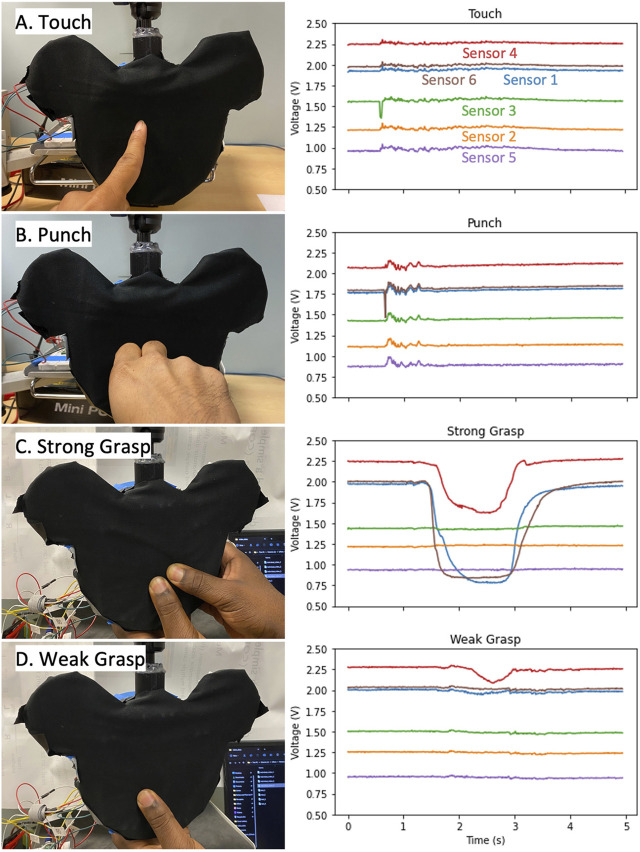
List of interactions collected with the corresponding sensor readings. Example videos of each interaction are provided in the Supplementary Material. **(A)** Touch **(B)** Punch **(C)** Strong Grasp **(D)** Weak Grasp.

**FIGURE 9:**
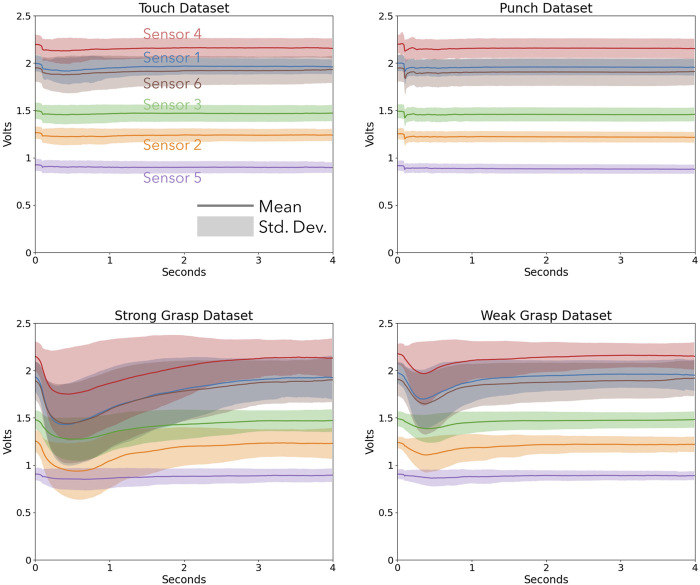
Plots explaining the dataset spread for each sensor and each interaction.

Collecting the sensor data across all trials yielded a data set of 2,480 interactions with distinguishable start times from Z score thresholding that could be aligned. This dataset was used to train the classification model and validate it. Figure 8 shows examples of the raw data collected from all 6 optical force sensors for Touch (Figure 8A), Punch (Figure 8B), Strong Grasp (Figure 8C) and Weak Grasp (Figure 8D). While not all sensors were engaged during every interaction, at least two sensors were activated. Figure 9 shows the spread of the dataset collected for the four interactions. Each figure shows the mean and standard deviation spread of all six sensor signals for each interaction. Although baseline voltage values read by each sensor differed, each had relatively stable voltage outputs and consistently detected interactions. The punch and touch interactions resulted in low voltage changes and involved all sensors. In contrast, both grasp actions resulted in moderate to large voltage changes and involved a subset of sensors. The similarity between sensor signals for an individual interaction can be observed in each subplot. The contrast between the signals for different interactions can be observed between different subplots, thus implying the use of classification algorithms to be valid.

**FIGURE 10:**
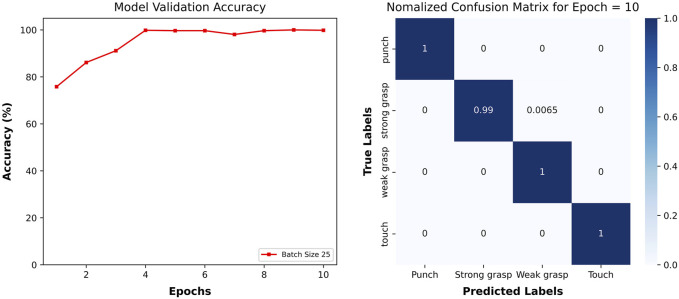
Performance of model trained on data from all six sensors.

The accuracy of the machine learning model during the validation stage depended primarily on the batch size and number of epochs specified for training (Supplementary Material). Choosing optimal values for batch size and number of epochs allows the model to achieve high classification accuracy while reducing computational cost. A hyperparameter sweep of batch sizes 5, 25, 50, and 100 was conducted. We found that a batch size of 25 achieved over 95% accuracy without incurring high computing cost (see Supplementary Figures S1, S2). Though accuracy provides a good representation of the overall performance of the model, it does not provide insight into how the model performs in classifying specific interactions from the data. To understand which interactions were being mistaken for others, a confusion matrix was created. The confusion matrix records all instances of classification and organizes them based on their true and predicted values. For easier interpretation, the confusion matrix is normalized and displayed as a heat map. For the 6-sensor-model training, consistent high accuracy was shown after the fourth epoch. This model excels not only in overall accuracy but also in the precision at categorizing each action. As evident in Figure 10, the normalized confusion matrix for the 6-sensor array predominantly features values of 1 along the diagonal for most action categories. This indicates a near-perfect accuracy in label predictions, affirming the model’s robustness across all categories.

After attaining a high accuracy for the trained model, we investigated whether a toy with only one sensor could provide sufficient data to the model. To investigate whether all six sensors were necessary in the device design for interaction classification, we trained six separate models corresponding to each sensor and its data. No other aspect of the models was changed from the original other than the “channels” parameter. It was found that in every case, the models using data from only one sensor were at least 20 percent less accurate. While the accuracy of the single-sensor models is lower than that of the 6-sensor model, their performance stabilizes with fewer fluctuations after several epochs. This suggests that the single-sensor models faced challenges in distinguishing between the interactions, which further motivates our proposed six sensor design.

**FIGURE 11:**
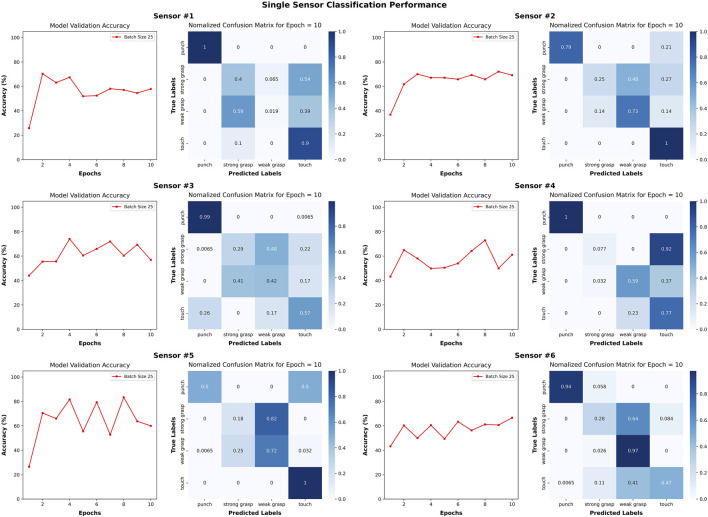
To motivate the six-sensor design of the PANDA toy, we investigate the interaction classification accuracy when only using a singe sensor. We trained six new models, one for each sensor, on the data only produced by that sensor. The validation set for each model is 620 samples. These plots show that the interaction classification accuracy suffers and the PANDA toy benefits from including all six sensors.

Notably, the misclassifications were not identical among single-sensor models. For instance, Sensor #6 frequently confused “weak grasp” actions with “touch” actions which seems to be counterintuitive. Such consistent challenges led to repeated errors in each validation, resulting in accuracy scores within a small range. Figure 11 presents the confusion matrices for individual sensors offering deeper insights into the single-sensor model’s misclassification of actions. Sensor #1, Sensor #2, and Sensor #6 exhibit a fluctuation of about 5% in accuracy over the last five epochs. Each of these sensors proficiently categorized punch with less error. This phenomenon can potentially be explained by the localized nature of certain actions on the toy. For instance, punch and touch actions induced vibrations throughout the toy, producing similar signals across all sensors. In contrast, grasp actions generated signals localized to the sensor most directly squeezed. Consequently, some actions may not have been discernible using data from a single sensor. From this experiment, we conclude that all six sensors in the toy are necessary for accurate interaction classification.

The work presented shows significant application in various haptic applications. As compared to other Smart Toys we show improvement in detection accuracy and robustness of interactions and location. By building a diverse dataset of infant interaction we envision the toy aiding clinicians in detecting developmental delays promptly and positively impacting life.

Other studies on tactile sensors used to collect human data have used machine learning models such as Neural Structured Learning (NSL) and modified Convolutional Neural Networks (CNN) to classify interactions Sun et al. (2017); Uddin and Soylu (2021); Jun and Choi (2020)). The model in this study was a ResNet, an off-the-shelf powerful neural network, trained using data collected from adults who were instructed to perform certain tasks. Since infants do not display collaborative behavior (Rihar et al., 2016), it would be time-consuming to prepare and label data (as with the data used in this study) from infants to retrain the model. Modifying the architecture of the model may allow flexibility in how data is presented to the model. This is why some studies have opted to use unsupervised models when dealing with data from non-collaborative subjects (Jun and Choi, 2020). However, using unsupervised learning often introduces uncertainty where it might not be as effective in isolating the behaviors of interest in such a varied dataset. The use of supervised learning is still favored over unsupervised learning since supervised learning allows for more targeted training on specific behaviors.

### 3.1 Limitations and design challenges

The custom lossy force sensors were not uniform in their performance when compressed. The inconsistent performance may have been due to the use of optic fibers at different lengths and our method of affixing the lattice structure to the Toy face using hot glue. For a sensor to cover more area, longer lengths of optic fiber were required. Looking at Figure 5C, Sensors 3 and 6 had the highest optic fiber lengths, Sensors 1 and 2 had shorter lengths, and Sensors 4 and 5 had the shortest. As optic fiber length increases, the traveling light has more length to escape, resulting in lower levels of light received and read by the sensors and, hence, lower baselines. Notably, Sensors 4 and 5 had differing baseline values while having the same optic fiber length. During the assembly process, glue got onto the optic fiber for sensor 5, attaching it permanently to the lattice and warping it. As a result, the baseline value shifted as a significant amount of light was continually lost—regardless, the sensor still provided similarly useful information, just scaled down. Additional calibration procedures are needed to understand the relationship between sensor response and sensor length. Noise was a feature in the data due to an experimental set-up that was not robust to vibrations. During human interaction trials, when subjects interacted with the toy, many of the external probes reacted and moved while being connected to the breadboard, resulting in electronic noise that’s visible in Figure 8 (prominent in touch and punch interactions; present in weak grasp interaction). In future iterations of the toy, all components will be housed internally and appropriate signal conditioning will be applied.

### 3.2 Future steps

Ideally, we will complete the sensorization of the entire toy using the lossy sensors and re-test with *in situ* experiments. The dataset generated and experiments conducted were in a simulated setup, Figure 6 very similar to the actual test setup with infants seen in Figures 1A, C. A clear next step is to build a more robust dataset with real data from infants. The dataset generated with adults is to show proof of concept of the sensor, the integrated toy and the confidence in the ML model to classify interactions. It is correct to assume that there will exist a bias if we use the same dataset with infants. The future goal is to build a completely new robust dataset with real infant interactions. The current classification algorithm was most accurate when all face 6 sensors were used to train it. This suggest that the placement of sensors on the toy will be critical to its success. Developing the algorithm to simultaneously classify whole body interactions as well as identify developmental risk in infants will be a near challenge. Additionally, we can investigate alternative algorithms specifically designed for temporal data.

The current method to classify interactions is to collect data from an entire trial and then obtain results after post-processing on a separate system, this can slow the analysis process greatly. Tiny Machine Learning (ML) is an upcoming field that deploys learning algorithms on the edge by running the inference on lightweight devices. Such a device can be housed in the toy and be used to get real-time results about infant-toy interactions. The infant-toy interactions can then be used to classify and alert clinicians on abnormalities in the interactions from a healthy infant to an infant at risk of neurological or motor developmental delays.

## 4 Conclusion

The work presented in this paper explains the development of a “Lossy Force Sensor” inspired by previous work in the field of optical fibers and soft robotics. Its characteristics to sense a wide force range with scalable lattice designs allow it to be used in many versatile applications. Here we have demonstrated its use in an infant toy that collects interaction data for analysis. The current version of the toy tested in this paper can be used to accurately identify interaction patterns using the lossy force sensors, however spatial information about where the interaction occurred is absent. Exploring how to increase this capability will be explored.

Like the previous version, the goal is to have a version of Ailu2 that incorporates lights, sound and additional kinematic sensors such as IMUs. While, the lossy force sensors by themselves provide ample tactile information that can be used for classification, incorporating IMUs into the toy will give even more rich data that can be used to improve results and learn insightful information. With the addition of lights and sounds the Ailu2 toy will provide feedback to the infants, which is often alluring to infants and encourages increased interactions.

The ML algorithm used was accurate in classifying the four different interactions that were performed on the toy. From a clinical perspective, this is helpful information as it aids the clinician’s assessment of the infant and qualitatively informs them about the number and kind of interactions performed during the trial. This allows the clinician to focus on more niche cues that infants show during trials that can help with their assessment and use the classified interaction results as a supplement to this.

## Data Availability

The raw data supporting the conclusion of this article will be made available by the authors, without undue reservation.
