# Supplementary material for: Towards an AI-driven soft toy for automatically detecting and classifying infant-toy interactions using optical force sensors
Source: Front Robot AI. 2024 Mar 12;11:1325296. doi: 10.3389/frobt.2024.1325296 (PMC10963494; doi:10.3389/frobt.2024.1325296)
Supplement: Supplementary file 2 [file DataSheet1.PDF]

Supplementary Material

1 SUPPLEMENTARY DATA

Four videos are included to demonstrate the real-time data output from the four different interactions (strong grasp, weak grasp, touch, punch) performed by a participant.

2 SUPPLEMENTARY FIGURES

These figures show the impact of the epoch and batch size hyperparameters on the model's accuracy in classifying trials, which informed the selection of the best-performing hyperparameter values.

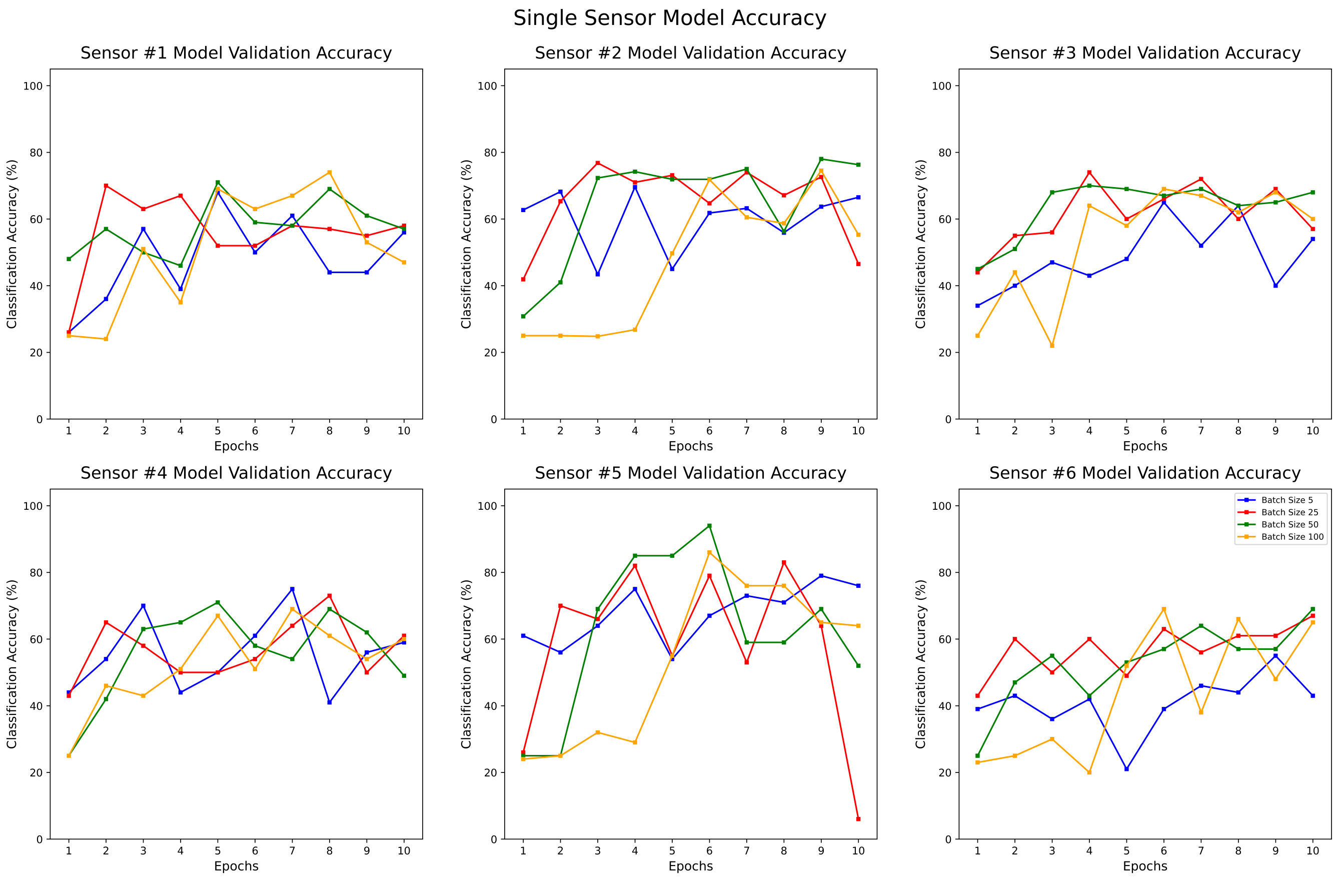

Figure S1. Classification accuracy of models trained on data from a single sensor

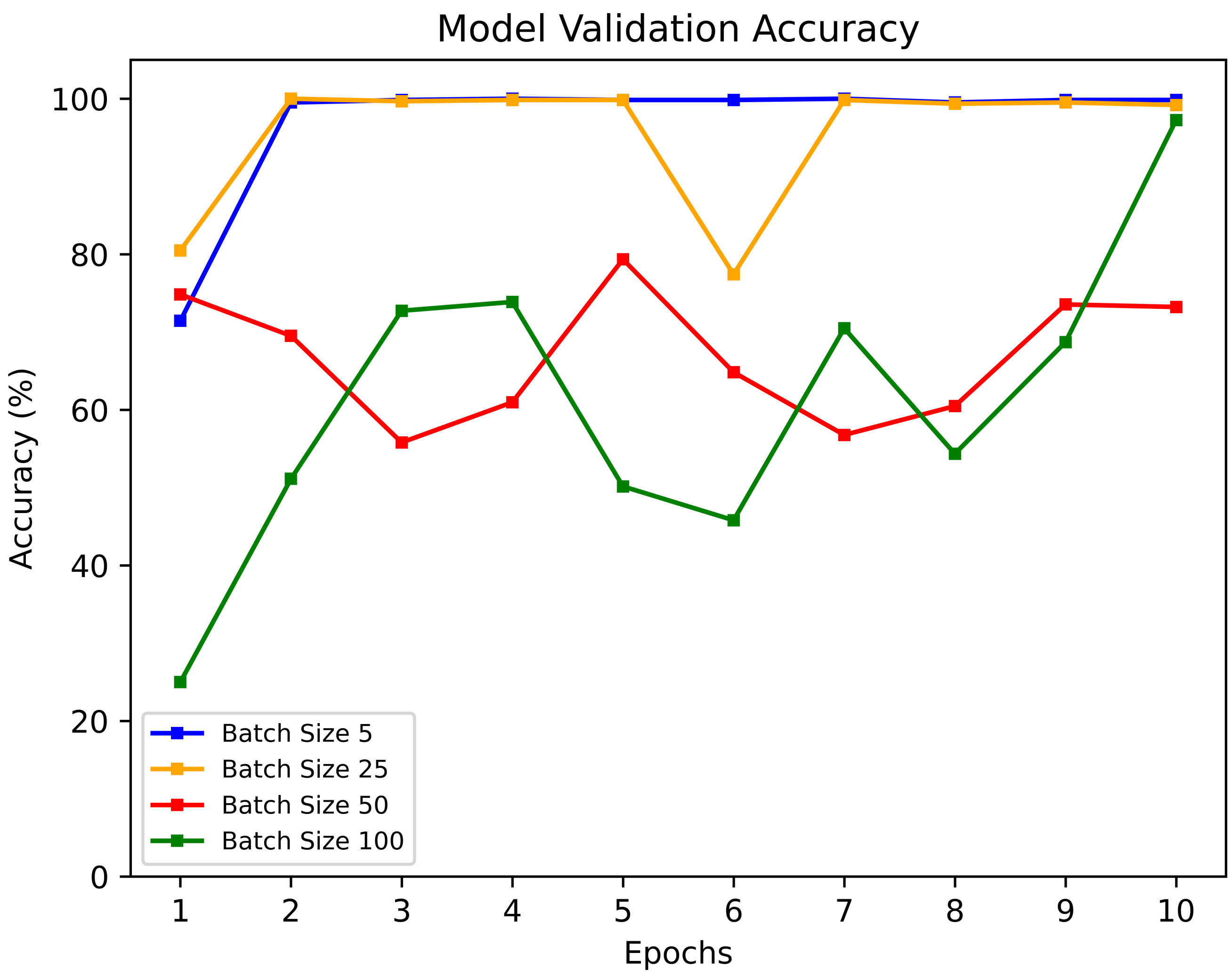

Figure S2. Accuracy of the 6-sensor-model trained with different batch sizes
